# Identifying Prognostic Criteria for Survival after Resuscitation Assisted by Extracorporeal Membrane Oxygenation

**DOI:** 10.1155/2016/9521091

**Published:** 2016-02-23

**Authors:** Alexandrine Brunner, Natacha Dubois, Peter C. Rimensberger, Oliver Karam

**Affiliations:** ^1^University of Geneva, Centre Medical Universitaire, 1 Rue Micheli-du-Crest, 1205 Geneva, Switzerland; ^2^Pediatric Intensive Care Unit, Geneva University Hospital, 6 Rue Willy Donzé, 1205 Geneva, Switzerland

## Abstract

To improve survival rates during CPR, some patients are put on extracorporeal membrane oxygenation (ECMO). Among children who have undergone ECMO cardiopulmonary resuscitation (ECPR), the overall rate of survival to discharge is close to 40%. However, despite its wide acceptance and use, the appropriate indications and organizational requirements for ECPR have yet to be defined. Our objective was to assess the clinical outcomes of children after ECPR and to determine pre-ECPR prognostic factors for survival to guide its indication. Among the 19 patients who underwent ECPR between 2008 and 2014 in our center, 16 patients (84%, 95% confidence interval: 62–95%) died during their hospital stay, including nine (47%) who were on ECMO and seven (37%) after successful weaning from ECMO. All three survivors had normal cognitive status, but one child suffered from spastic quadriplegia. Survivors tended to have lower lactate, higher bicarbonate, and higher pH levels before ECMO initiation, as well as shorter length of resuscitation. In conclusion, in our center, ECPR has a poorer outcome than expected. Therefore, it might be important to identify, a priori, patients who might benefit from this treatment.

## 1. Introduction

Since the 1980s, extracorporeal membrane oxygenation (ECMO) has been used during cardiopulmonary resuscitation (CPR) to improve outcomes in selected patients, as a bridge to cardiac recovery or transplantation. According to the international registry of the Extracorporeal Life Support Organization (ELSO), more than 2370 children have benefited from ECMO-assisted CPR (ECPR) since its introduction [1].

The 1-year survival rate has been reported to be less than 5% for children after CPR lasting more than 30 minutes for out-of-hospital arrests [2]. Consequently, ECPR is used in cases requiring prolonged times of chest compressions. As a result, the rate of survival to hospital discharge after ECPR in children is between 41% [1] and 49% [3]. It has therefore been suggested that ECPR improves survival after prolonged circulatory arrest. However, the survival-to-discharge rate of children after in-hospital circulatory arrest without ECPR ranges from 39% [4] to 49% [5]. Thus, the effect of ECPR is not completely clear.

Some authors have assessed predictors of survival after ECPR. For example, Joffe et al. found that the most consistent predictors of mortality were noncardiac disease, renal dysfunction during ECMO, neurological complications during ECMO, and lowest pH during ECMO. Duration of CPR before ECMO was a predictor of survival in two studies but was not a predictor in nine other studies [3]. However, these predictors are not measured before ECMO cannulation and cannot aid the clinician in deciding, a priori, whether such a treatment should be offered. Therefore, the objectives of our study were to evaluate the clinical outcome after ECPR at our institution and to determine the factors associated with the rate of survival to hospital discharge.

## 2. Methods

This report describes a retrospective study performed in the 12-bed tertiary pediatric Intensive Care Unit (PICU) at Geneva University Hospital from January 2008 to December 2014. All consecutive children (<16 years old) at this institution who underwent at least 1 minute of chest compressions during the 15 minutes before initiation of ECMO flow were included in the study. This study was approved by our ethics review board, which waived the need for individual consent provided that the report did not allow the identification of specific patients.

The primary outcomes were survival after ECMO weaning and survival to hospital discharge, and secondary outcome was neurological status of hospital survivors, as assessed by the Pediatric Cerebral Performance Category (PCPC) score [6]. Demographic data, reason for PICU admission, comorbidities, cause of circulatory arrest, poorest results of laboratory tests before ECMO initiation, CPR duration until ECMO initiation (defined as the time when oxygenated blood was delivered to the patient through the ECMO cannulas), ECMO cannulation site (central or peripheral), ECMO duration, length of stay in the PICU after successful ECMO weaning, and cause of death were recorded.

Each year, an average of 600 critically ill children are admitted to our PICU, including 200 children who are admitted after cardiac surgery. More than three-quarters of these cardiac surgery patients are humanitarian patients from African countries, who are often older due to delayed diagnosis. Since 1996, between 6 and 12 ECMO runs per year have been performed in our PICU, with a reported survival rate of 69% [7]. ECMO cannulation is always performed by cardiac surgeons. Neither the cardiac surgeons nor the specialized perfusion technicians are in-house during nights and weekends. Our institution does not offer dry or clear-primed ECMO circuits at all times. Our PICU does not provide recommendations on the indications for ECPR. Therefore, the experience with ECPR at this institution was reviewed to develop such guidelines.

Our PICU does not use hypothermia after cardiac arrest but actively avoids hyperthermia in these patients.

Due to the relative rarity of ECPR, no sample size was calculated. To permit homogeneity of data, the data were collected over a 7-year interval (the electronic Patient Data Monitoring System was installed at our PICU in 2008).

Descriptive statistics are reported as the mean ± standard deviation (SD), median (interquartile range, IQR), or proportions with their 95% confidence intervals (CIs). The Kruskal-Wallis test was used to assess the association between survival status (death on ECMO, death after ECMO weaning, or survival to hospital discharge) and continuous variables (laboratory tests or CPR length before ECMO initiation). A Chi-square test was used to assess the association between survival status and dichotomous variables (cardiac or noncardiac patients). All tests were two-sided, with an alpha level of 0.05. All statistical analyses were performed with SPSS version 20 for Mac (SPSS, Chicago, IL, USA).

## 3. Results

Between 2008 and 2015, 51 patients underwent ECMO in our unit (average of 7.3 ECMO runs per year). Of these patients, 19 (37%) were put on ECMO during resuscitation. Their median age was 44 months (IQR 14; 111) and their median weight was 19 kg (IQR 7; 25). Medical conditions that lead to ECPR included cardiac surgery (11/19, 58%), dilated cardiomyopathy (3/19, 18%), septic shock (2/19, 11%), circulatory arrest of unknown origin (2/19, 11%), and primary pulmonary hypertension (1/19, 5%).

No patients require CPR in the 12 hours prior to the episode leading to ECPR. All patients required continuous chest compression until ECMO initiation, as none presented with a return of spontaneous circulation, even briefly, prior to ECMO initiation. The median duration of CPR before ECMO initiation was 77 minutes (IQR 22; 120). Three patients had very short duration of CPR (<10 minutes). These were already in the process of ECMO cannulation, prior to cardiac arrest and CPR. Only one patient suffered from a witnessed but out-of-hospital arrest. His duration of CPR prior to ECMO initiation was 180 minutes.

Nine patients (47%) received ECPR during working hours. The length of CPR prior to ECMO initiation was not significantly shorter than those who arrested after working hours (64 versus 91 minutes, *p* = 0.39).

All ECMOs were venoarterial. Peripheral cannulation was performed in 10 patients (53%); no limb ischemia or other cannulation complications were observed. The median ECMO duration was 5 days (IQR 1; 11, Table 1).

Of the 19 patients included in this study, only three survived to hospital discharge (16%, 95% CI 6–38). All three survivors had normal cognitive status at hospital discharge. However, one patient suffered from spastic quadriplegia. All three survivors had preexisting cardiac diseases (complex congenital malformations for two patients and idiopathic pulmonary hypertension for one). The latter required a lung-heart transplant.

Nine children (47%, 95% CI 27–68) died while on ECMO, including 4 patients (44%) who were brain dead, 2 patients (22%) who died of multiple organ failure despite support, 1 patient (11%) who died of refractory fungal septic shock, and 1 patient (11%) who died of refractory heart failure without being eligible for heart transplant. Life support was withdrawn for 1 patient (11%) due to severe hypoxic-ischemic brain injury.

Seven children (37%, 95% CI 19; 59) died after successful ECMO weaning. The median time between ECMO weaning and death was 10 days (IQR 1; 13). Three patients (43%) presented a second circulatory arrest and were not successfully resuscitated, 1 patient (14%) died of refractory heart failure without being eligible for heart transplant, and 1 patient (14%) died of multiple organ failure. Life support was withdrawn for 2 patients (29%) due to severe hypoxic-ischemic brain injury.

**Figure 1:**
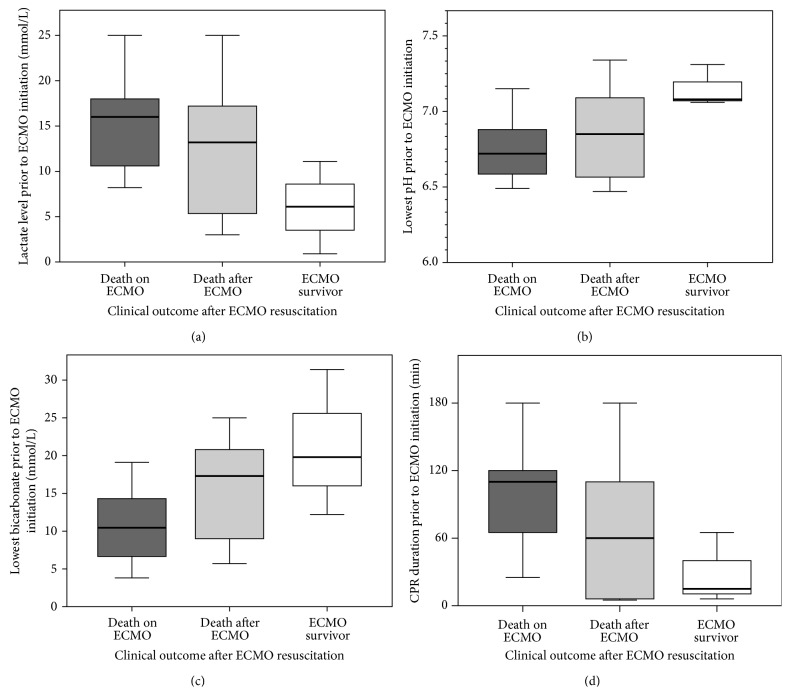
Boxplot of lactate (a), pH (b), and bicarbonate (c) levels before initiation of extracorporeal membrane oxygenation (ECMO) and cardiopulmonary resuscitation (CPR) duration (d) until ECMO initiation, according to clinical outcome (death on ECMO in dark gray, death after successful ECMO weaning in light gray, and survival rate to hospital discharge in white).

Higher lactate, lower pH, and lower bicarbonate levels before ECMO cannulation, as well as longer durations of CPR before initiation of ECMO flow, showed nonsignificant trends towards an association with mortality (Table 1, Figure 1).

## 4. Discussion

This retrospective study shows a low survival rate among children who had ECPR at our center. Our data also suggest that higher lactate, lower pH, and lower bicarbonate levels before ECMO cannulation, as well as longer duration of CPR before initiation of ECMO flow, might be associated with poorer clinical outcomes.

Several studies have evaluated survival after ECPR. The ELSO registry reported a 41% (976/2370) rate of survival to hospital discharge in the pediatric population [1]. Our lower survival rate might be explained by our specific case-mix, by our ECPR team organization, or by our indications for ECPR. As most of our cardiac surgery patients are humanitarian patients from African countries, their malformations might be more complex which might affect their chances of return of spontaneous circulation. Unfortunately, it is not possible to assess the importance of the case-mix properly, as there are no comparative data on the exact diagnosis and comorbidities in the ELSO database. Furthermore, because most studies of ECPR have not identified the duration of CPR before ECMO initiation as a risk factor [3], the fact that our ECPR team is not in-house at all times might not entirely account for our increased mortality rate. Our results might also be explained by our current indications, as some patients were put on ECMO after more than 1 hour of CPR and lactate levels higher than 10 mmol/L. Unfortunately, there are currently no solid data to indicate, a priori, which patients would not benefit from ECPR.

Our study did not have the power to assess robustly the risk factors associated with worse outcome. However, preexisting cardiac disease, longer CPR, higher lactate, and lower bicarbonate and pH levels might be associated with worse clinical outcome. Although not statistically significant, these results seem to have a solid physiological basis: lower oxygen delivery during prolonged CPR would induce higher levels of lactate, which is produced during anaerobic metabolism. The higher lactate levels, in turn, would decrease the bicarbonate and pH levels. Similar results have been found among cases of in-hospital circulatory arrest without ECPR [4]. Unfortunately, similar data are not available from the international ELSO registry, as they are not systematically collected. Considering the lack of evidence regarding prognostic factors measured before ECMO initiation, we believe that further studies are urgently required to help physicians decide which patients should or should not be put on ECMO during CPR.

Certain limitations of this study must be recognized. First, the small sample size precludes the assessment of specific risk factors. However, the CI values allowed us to conclude that our survival rate was significantly lower than that of larger series. Second, the retrospective design might introduce errors in the estimation of some variables. However, this issue should not be relevant for the outcomes we chose, which are not subject to recollection errors. Third, our results might not be generalizable to other centers, as our case-mix and ECPR team organization are hospital-specific. Fourth, systematic CT scans were not obtained in all patients who died on ECMO due to brain death. Therefore, there is a possibility that the ECMO per se was responsible for the unfavorable outcome, due to cannulation or circuit-related embolic events. Finally, there was no consensus on the indication to ECPR in our PICU, which might explain the long CPR time prior to ECMO initiation.

Our results raise important ethical questions. Is it appropriate to continue offering ECPR, given our high mortality rate? Considering that the expected survival rate might have been lower than 5% without ECMO due to prolonged CPR duration, the three survivors with intact cognitive functions clearly seemed to have benefited from ECPR. Moreover, one-third of the nonsurvivors were diagnosed with brain death, and their families were offered the possibility to donate their organs, hence potentially saving other lives. However, the economic burden of ECPR should not be overlooked, and a better selection of patients should be actively sought.

In conclusion, survival after ECPR is low at our center. Other studies are needed to determine the prognostic factors for survival before ECMO initiation, to identify patients who would be most likely to survive after ECPR.

## Figures and Tables

**Table 1:** Demographic data, laboratory tests before ECMO initiation, and CPR duration, according to survival status.

Variable	Death on ECMO	Death after ECMO weaning	Survival to discharge^*∗*^	*p* value^†^
	(*n* = 9)	(*n* = 7)	(*n* = 3)	
Age (months)	57 (27; 100)	37 (9; 110)	111 (14; 182)	0.42
Weight (kg)	20 (12; 25)	9 (6; 20)	25 (9; 55)	0.16
Noncardiac disease	4 (44%)	0 (0%)	0 (0%)	0.06
ECMO duration (days)	4 (2; 6)	6 (1; 7)	11 (1; 14)	0.56
Highest lactate level before ECMO (mmol/L)	16 (9.45; 18)	13.2 (3.3; 20)	6.1 (0.9; 11.1)	0.12
Highest potassium level before ECMO (mmol/L)	5.8 (4.2; 8.5)	4.6 (4.4; 5.3)	4.3 (4.1; 4.8)	0.29
Lowest pH level before ECMO	6.72 (6.55; 6.89)	6.85 (6.55; 7.21)	7.08 (7.06; 7.31)	0.16
Highest pCO_2_ level before ECMO (kPa)	10.4 (6.4; 14.1)	8.1 (4.6; 13.8)	8.7 (5.8; 9.8)	0.43
Lowest bicarbonate level before ECMO (mmol/L)	10.5 (5.5; 14.8)	17.3 (6.3; 23.5)	19.8 (12.2; 31.4)	0.13
CPR duration until ECMO initiation (minutes)^‡^	110 (52; 120)	60 (5.5; 145)	15 (6; 65)	0.17

^*∗*^Survival to discharge was defined as survival to hospital discharge.

^†^The statistical test assessed the differences between the three groups (death on ECMO, death after ECMO weaning, survival to discharge).

^‡^We considered ECMO initiation as the time when oxygenated blood was delivered to the patient through the ECMO cannulas.

Data are shown as the median (interquartile range) or the number (%). ECMO: extracorporeal membrane oxygenation; pCO_2_: partial pressure of carbon dioxide; CPR: cardiopulmonary resuscitation.
